# Multi-Lineage Differentiation of Human Umbilical Cord Wharton’s Jelly Mesenchymal Stromal Cells Mediates Changes in the Expression Profile of Stemness Markers

**DOI:** 10.1371/journal.pone.0122465

**Published:** 2015-04-07

**Authors:** Hamad Ali, Majda K. Al-Yatama, Mohamed Abu-Farha, Kazem Behbehani, Ashraf Al Madhoun

**Affiliations:** 1 Department of Basic Science Research, Dasman Diabetes Institute, 1180 Dasman, Kuwait; 2 Department of Medical Laboratory Sciences (MLS), Faculty of Allied Health Sciences, Health Sciences Center, Kuwait University, Kuwait City, Kuwait; 3 Dar Al Baraa Medical Center, 3357 Hawally, Kuwait; 4 Biochemistry and Molecular Biology Unit, Dasman Diabetes Institute, 1180 Dasman, Kuwait; French Blood Institute, FRANCE

## Abstract

Wharton’s Jelly- derived Mesenchymal stem cells (WJ-MSCs) have gained interest as an alternative source of stem cells for regenerative medicine because of their potential for self-renewal, differentiation and unique immunomodulatory properties. Although many studies have characterized various WJ-MSCs biologically, the expression profiles of the commonly used stemness markers have not yet been addressed. In this study, WJ-MSCs were isolated and characterized for stemness and surface markers expression. Flow cytometry, immunofluorescence and qRT-PCR analysis revealed predominant expression of CD29, CD44, CD73, CD90, CD105 and CD166 in WJ-MSCs, while the hematopoietic and endothelial markers were absent. Differential expression of CD 29, CD90, CD105 and CD166 following adipogenic, osteogenic and chondrogenic induction was observed. Furthermore, our results demonstrated a reduction in CD44 and CD73 expressions in response to the tri-lineage differentiation induction, suggesting that they can be used as reliable stemness markers, since their expression was associated with undifferentiated WJ-MSCs only.

## Introduction

In recent years, the biological and clinical interest in Mesenchymal stem cells (MSCs) has increased noticeably due to their unique stemness characteristics. MSCs are non-hematopoietic cell population with multipotent precursor properties which has high degree of self-renewal and exhibit multi-lineage differentiation potential [1]. Although, MSCs reside primarily in the bone marrow, where they were first characterized [2]; studies have shown broad post-natal organ distribution of MSCs compartment including brain, liver, kidney, lung, adipose and connective tissues [3], as well as fetal tissues such as placenta, umbilical cord blood and matrix [4, 5] Unlike embryonic stem cells, the use of MSCs for clinical applications is ethically acceptable and no risk is associated with teratoma formation [6].

MSCs are described as immunologically privileged cells, modulate immune responses and exhibit anti-inflammatory properties (best reviewed in [7, 8]). MSCs lack the expression of the co-stimulatory surface antigens CD40, CD86 and CD80 that mediate T-cell activation [9–11] and suppress stimulated T-cells by activating TNF-a/NF-kB signaling pathway [12]; and/or secreting soluble factors such as Eph/ephrin [13], prostaglandin E_2_ [14] or indoleamine 2,3-dioxygenase [15]. MSCs drastically inhibit B-cell proliferation, differentiation and chemotactic behavior [16]. MSCs restrain the proliferation, maturation and activation of the innate immune system components, natural killer and dentritic cells. In the presence of MSCs, the secretory cytokine profile and molecules related to antigen presentation of these cells are inhibited [17, 18]. Thus, the recipient immunological tolerance to the administration of MSCs makes them ideal for clinical practice and good potential for cell therapy.

Currently, studies are focusing on adult bone marrow as a source for MSCs that suffers from a number of clinical limitations such as invasive collection procedures, the availability of suitable cell donors, poor mobility, limited long-term proliferation potential and age-limited frequency and differentiation capacity [19, 20]. Accordingly, there is a need to find other source of MSCs that possess similar characteristics of bone marrow MSCs but conquer these limitations.

**Fig 1:**
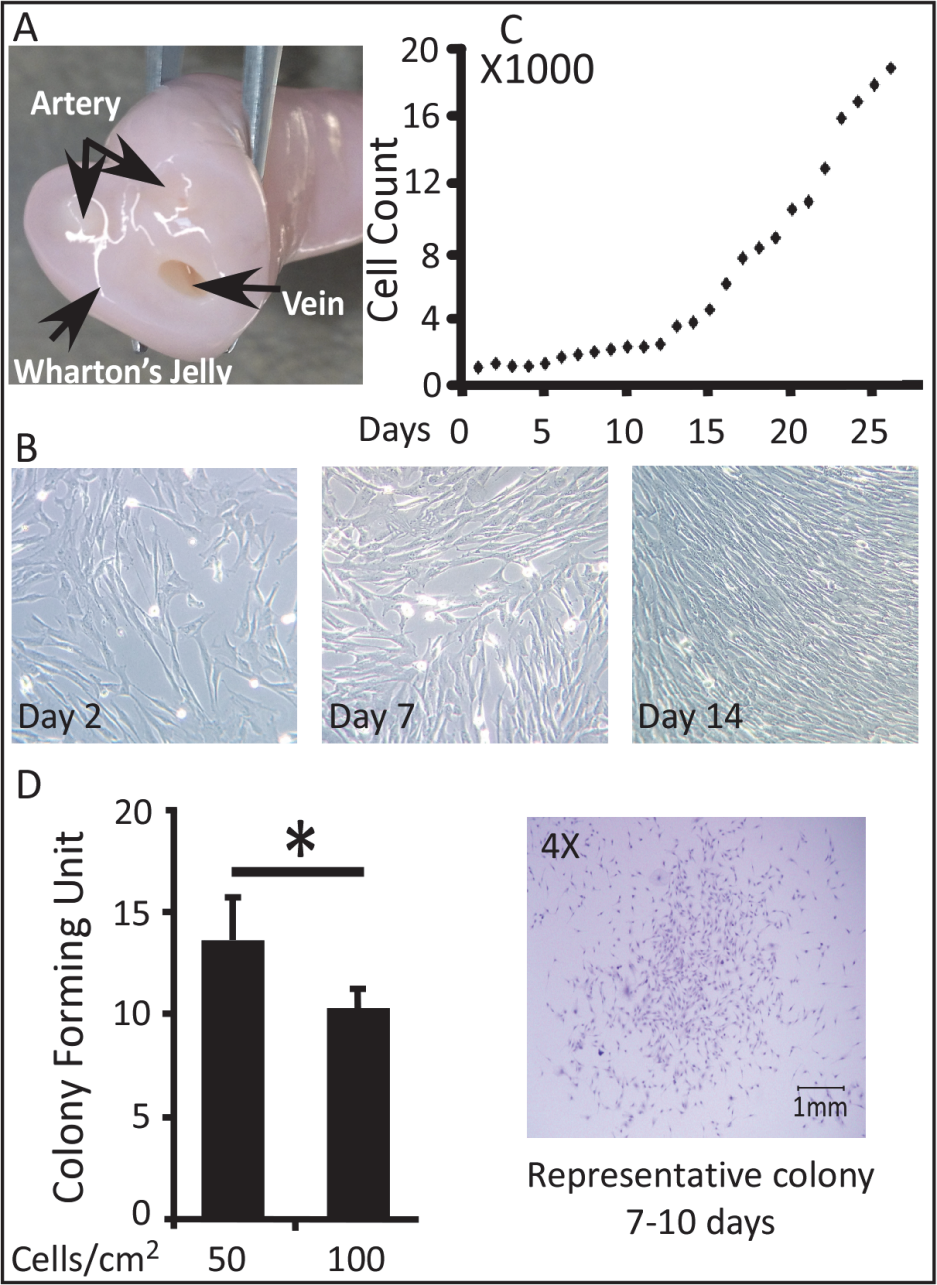
Source, Morphology and Growth Kinetics of WJ-MSCs. (A) The umbilical cord compartments. (B) Representative phase-contrast images of WJ-MSCs at Day 2, 7 and 14; 400X magnifications. (C) Growth rate, WJ-MSCs were seeded at a density of 1000 cells/cm^2^, cellular proliferation was detected in a daily bases. (D) CFU-F, cells were plated at a density of 50 or 100 cells/cm^2^ for 7–10 days. CFU-F was determined by (number of colonies formed/number of cells inoculated) x 100. *P < 0.05; the significance was evaluated by Student’s *t-*test.

Human umbilical cord blood (UB-MSCs) and Wharton’s jelly (WJ-MSCs) stem cells are conventional model of choice for the development of potential novel cellular therapies (Fig 1A). Similar to adult MSCs, these cells acquire the stemness defined characteristics including multipotent differentiation potential, specific surface antigen expression and adherence to plastic [21]. Both UB- and WJ-MSCs are easy to collect from umbilical cord, which is considered as a medical waste, with painless noninvasive isolation procedure and no associated ethical constraints [22–24]. Although, a large donor pool is available, UB-MSCs are less attractive for clinical application due to their low frequency, poor proliferation rate and culture limitations [6].

WJ-MSCs are myofibroblastoid stromal cells isolated from the gelatinous layer within the umbilical cord tissue. The young WJ-MSCs are proliferative, immunosuppressive and remarkably stable under cultural conditions [25, 26]. Gene expression profiling studies revealed that WJ-MSCs share molecular signature similar to that of embryonic stem cells [27]. Relative to adult MSCs, a higher expression of the pluripotency markers like NANOG, Oct 3/4 and Sox2 were observed in cultured WJ-MSCs [28–30].

WJ-MSCs do not express a unique surface marker but rather express several markers that determine their identity as described by the guidelines recommendations of the International Society for Cellular Therapy (ISCT) for the characterization of MSCs [21]. WJ-MSCs are positive for surface antigens including the cell adhesion receptors, integrin β1 (CD29 [31]) and the homing receptor (CD44, hyaluronan receptor [32]); the GPI-anchored protein, ecto-5’-nucleotidase (CD73 [33]); and thy-1 (CD90 [34]), signal transduction molecules and mediators of cell-cell and cell-matrix interactions; the intercellular adhesion molecule-1, ICAM-1 (CD54 [35]); TGF-B receptor binding glycoprotein, endoglin (CD105 [36]); the activated leukocyte cell adhesion molecule, ALCAM (CD166 [37]); the decay-accelerating factor (CD55 [35]), and the type II integral membrane protein (CD13 [35]). Further, WJ-MSCs are negative for the expression of the hematopoietic surface antigens CD14, CD45, CD34 and the endothelial markers CD106 and CD133 [1, 25, 38].

The aim of this study was to isolate and characterize WJ-MSCs from umbilical cords of end-term birth. A detailed description of the stem cell plasticity, their capacity to differentiation into adipogenic, osteogenic and chondrogenic lineages and the associated markers during the differentiation process was studied. For the first time, we explored WJ-MSCs stemness surface markers differential profile during the multi-lineage differentiation procedures.

## Materials and Methods

### Ethic Permission and Procurement of Human Samples

The study was pre-approved by the Ethical Review Committee at the Dasman Diabetes Institute (protocol No: 2013–009) in accordance with the World Medical Association Declaration of Helsinki- Ethical Principles for Medical Research Involving Human Subjects. Human umbilical cords were collected from a full-term natural delivery healthy infants after obtaining written informed consents from the mothers (age 20–30 years old) and family using the guidelines recommended by the Ethical Review Committee. Umbilical cords (5–10 cm in length) were collected immediately after birth in sterile surgical tubes, transported to the laboratory, stored at -40°C and processed within 18 hrs.

### Isolation and Culture of WJ-MSC from Human Umbilical Cords

WJ-MSC were isolated and cultured as described [39–41] with modifications. Umbilical cords were washed three times with phosphate buffer saline (PBS) containing penicillin (200 units/ml), streptomycin (200 μg/ml) and amphotericin B (5 μg/ml) to remove blood cells. Then, the umbilical cords were cut into 2–3 cm pieces, and each piece was transected longitudinally and the blood vessels were dissected. Wharton’s jelly tissue was diced into small pieces and transferred into 15 ml tubes containing DMEM/Hams’s F-12 (1:1 vol/vol) culture medium supplemented with 10% MSC-qualified FBS, collagenase type B (1 μg/ml), penicillin (100 units/ml), streptomycin (100 μg/ml), and amphotericin-B (2.5 μg/ml); cell culture media and supplements are purchased from Invitrogen. The tissue was digested at 37°C in a gentle orbital shaker until a tissue homogenate was obtained in approximately 4 hrs. The homogenate was centrifuged 500g for 20 minutes. The cell pellet was suspended in the described culture medium containing 10 ng/ml basic fibroblast growth factor (βFGF, R&D Systems), and with no collagenase. Cell proliferation was monitored during the first expansion period passage 0, (P0), for several days before the first passage (P1). Upon reaching 100% confluence, cells were detached using 0.05% trypsin / 0.02% EDTA in PBS for freeze down and further subcultures.

### Growth Kinetic Characteristics

Growth kinetics experiments were performed as described [30, 40, 42]. The proliferation curve and the accumulative cell population doubling time were determined by culturing the cells to 80% confluence, harvesting, and counting at the indicated time points. Growth kinetics was evaluated by calculating accumulative population doubling time. Population doublings were calculated using the formula: X = [log10(Nh)-log10(Ni)]/log10(2); where X is the population doubling; Ni is the inoculum number and Nh is the cell harvest number. The cumulative population doubling level was calculated by adding the population doubling for each passage to the population-doubling level of the previous passage. The population-doubling time was obtained by the formula: TD = [t*log2]/[log(Nh)–log(Ni)]; where Ni is the inoculum cell number, Nh is the cell harvest number, and t is the time of the culture (in hours) [35].

### Colony Forming Unit-Fibroblast (CFU-F)

CFU-F assays were performed as previously described [43] with some modifications. WJ-MSCs (P2- P5 from 3 different umbilical cord sources; 9-replicates each) were cultured at approximate densities of 50 and 100 cells/cm^2^ in 6 well plates. After 7–10 days, culture media was removed; cells were washed with 1X PBS and fixed with ice-cold methanol for 5 min. The plates were air dried, stained with 0.4% Trypan blue solution (Invitrogen) to be counted. The amount of colonies (1–8 mm in diameter) ware established by scoring individual colonies derived from a single precursor using Stereo-microscope (Olympus, SDF plapo 1XPF). The plating efficiency was determined by the formula: (number of colonies formed/number of cells inoculated) X 100.

### Flow Cytometry Analysis

A small fraction of Day 0, undifferentiated WJ-MSC, cells (passage 2–4, 10^5^ cells) from at least 3 different preparations were analyzed using Flow cytometry (BD LSRII) for the expression of MSC surface markers using specific antibodies (Table 1) as described previously [44]. After addition of antibodies according to recommended concentrations, tubes were incubated in the dark at room temperature for 20 minutes; flow cytometry analysis was performed and then data analysis was done using BD FACSDiva software.

**Table 1:** Primary Antibodies used for Flow Cytometry characterization of undifferentiated cells.

Antibody	Conjugate	Manufacturer	Catalogue number
CD29	AL700	Biolegend	303019
CD34	PE-Cy7	Biolegend	343515
CD44	Pacific Blue	Affymetrix eBioscience	48–0441
CD45	APC-Cy7	Biolegend	103115
CD73	Pacific Blue	Biolegend	344011
CD90	Fitc	Biolegend	328107
DC105	APC	Biolegend	323207
CD106	PE-Cy5	Biolegend	305808
CD133	APC	Miltenyl Biotec	130-098-829
CD166	Fitc	AbD Serotec	MCA1926F

### Western Blot and Immunofluorescence Assay

Western Blot assay was performed as described previously [45, 46]. Immunofluorescence assays were performed on undifferentiated- and adipogenic-differentiated WJ-MSC. WJ-MSCs differentiated into adipocytes were first Oil red-O stained as described below and then subjected to immunefluoresces assay. Cells were fixed with 4% paraformaldehyde for 15 min, washed extensively with PBS and then incubated overnight with the antibodies. Anti human-CD29 (R&D Systems) were conjugated with Alexa Fluor 594; were as anti human-CD44 (Novus-bio),-CD90 (R&D Systems) and-CD166 ((R&D Systems) were conjugated with Alexa Fluor 488 using APEX Antibody Labeling Kits (Invitrogen). Pre-conjugated anti-human CD105-PE (clone SN6), anti-human CD73-PE (clone AD2) were purchased from eBioscience. Fluorescent and phase contrast images were captured using Confocal Laser-Scanning microscope (Zeiss, LSM 710) as previously described [47].

### RNA Extraction, cDNA Synthesis and qRT-PCR Reactions

Total RNA was extracted from cells using the total RNA purification kit (Norgen Biotek, Canada) following the manufacturer's protocol. First strand cDNA was synthesized from 50–100 ng RNA by reverse transcription using QuantiTect Reverse Transcription Kit (Qiagen Inc., USA). qRT-PCR reactions were performed as described [46, 48]. Primer pairs (Table 2) were selected from PrimerBank [49] and tested for equivalent efficiency. qRT-PCR was performed on the ABI7900 system (Applied Biosystems, USA) using SDS software. Relative gene expression was calculated using comparative Ct method as previously described [45, 50]. Results were normalized to GAPDH, and averages ± SEM are shown expressed relative to Control or Day 0 undifferentiated cells, as indicated.

**Table 2:** Oligonucleotide sequences of primers utilized for real-time qRT-PCR.

Genes	Forward Primer (5’-3’)	Reverse Primer (5’-3’)
CD29	GTAACCAACCGTAGCAAAGGA	TCCCCTGATCTTAATCGCAAAAC
CD34	AATCAGCACAGTGTTCACCAC	TGCCCTGAGTCAATTTCACTTC
CD44	CTGCCGCTTTGCAGGTGTA	CATTGTGGGCAAGGTGCTATT
CD45	ATTACCTGGAATCCCCCTCAAA	TTGTGAAATGACACATTGCAGC
CD73	GCCTGGGAGCTTACGATTTTG	TAGTGCCCTGGTACTGGTCG
CD90	ATCGCTCTCCTGCTAACAGTC	CTCGTACTGGATGGGTGAACT
CD105	TGCACTTGGCCTACAATTCCA	AGCTGCCCACTCAAGGATCT
CD106	GGGAAGATGGTCGTGATCCTT	TCTGGGGTGGTCTCGATTTTA
CD133	GGCCCAGTACAACACTACCAA	ATTCCGCCTCCTAGCACTGAA
CD166	ACTTGACGTACCTCAGAATCTCA	CATCGTCGTACTGCACACTTT
GAPDH	GGAGCGAGATCCCTCCAAAAT	GGCTGTTGTCATACTTCTCATGG
PPARg2	ACCAAAGTGCAATCAAAGTGGA	ATGAGGGAGTTGGAAGGCTCT
Adiponectin	AACATGCCCATTCGCTTTACC	TAGGCAAAGTAGTACAGCCCA
Osteoprotegerin (OPG)	GTGTGCGAATGCAAGGAAGG	CCACTCCAAATCCAGGAGGG
Osteopontin	CTCCATTGACTCGAACGACTC	CAGGTCTGCGAAACTTCTTAGAT
Chondroadherin (CHAD)	GGACCACAACAAGGTCACTGA	GTGGAATTTGGCGAGGTTCTC

### Proteomic Analysis

Proteomics were analyzed using LTQ-Orbitrap velos as described previously [51]. Briefly, peptides were suspended in 5% formic acid and then loaded on a C18-A1 easy column for desalting (Proxeon Biosystems, Denmark). Desalted peptides were than directed to a C18-A2 analytical easy column and eluted at a gradient of 5 to 35% acteonitrile with 0.1% formic acid for 120 min (Proxeon Biosystems, Denmark). The full MS spectra scan was performed at a resolution of 60,000. MS/MS spectra were acquired in a data-dependant acquisition mode selecting top 20 spectra. Raw data files were analyzed using Maxquant 1.3.0.5 software (Thermo Scientific; Germany) using Sequest and Mascot search engine against the Homo sapiens International Protein Index (IPI) protein sequence database version 3.68 (European Bioinformatics Institute, United Kingdom).

### WJ-MSC *in vitro* cellular differentiation

The *in vitro* lineages differentiation was performed on WJ-MSC (P2-P5) isolated from three different pregnancies and each differentiation experiment was done in triplicate, as described by Wang *et al*., 2004, with modifications [52]. For RNA extractions and time point differentiation profile, cells were harvested on weekly bases till the end of each experiment.

#### Adipogenic differentiation

A 24 well plate was cultured with 2x10^4^ cells/well. At 50–60% cell confluency, cells were treated with Adipogenic medium contained DMEM-low glucose supplemented with 10% FBS, penicillin (100 units/ml), streptomycin (100 μg/ml), 1μM dexamethasone, 500 μM isobutylmethylxanthine (IBMX), 5 μg/ml insulin, 200 μg/ml ascorpate-2-phosphate. The Culture medium was replaced every 3 days for 4-weeks period. At the end of the experiment weeks 3; cells were fixed in 4% paraformaldehyde for 15 min at room temperature, washed with sterile water and then with 60% isopropanol, finally, stained with 0.5% Oil red-O (Sigma) in isopropanol (wt/vol) for 30 min. Excess stain was removed and the cells were washed 3 times with sterile water. Control cells were gown in culture medium without inductive supplements.

#### Osteogenic Differentiation

A 24 well plate was cultured with 2x10^4^ cells/well. At 80% cell confluency, cells were treated with osteogenic medium contained DMEM-low glucose supplemented with 10% FBS, penicillin (100 units/ml), streptomycin (100 μg/ml), 100 nM dexamethasone, 10 mM β-glycerophosphate and 50 μg/ml ascorbate-2-phosphate. The Culture medium was replaced every 3 days for 3-week period. Cells were fixed and osteogenesis was detected using Alkaline phosphatase detection Kit (Millipore) following the manufacturer protocol. Control cells were gown in culture medium without inductive supplements.

#### Chondrogenic Differentiation

The protocol, described by Karahuseyinoglu *et*. *al*.[53], was followed with some modification. 4x10^5^ cells were centrifuged 1000 rpm for 10 minutes in 15 mL tubes. Chondrogenic medium was gently added without disturbing the cells pellet. Cells were allowed to form aggregates by incubating the tubes at 37°C in 5% CO2 in a humidified environment. Chondogenic medium contains DMEM-high glucose supplemented with 20 ng/ml TGF-β3, 100 nM dexamethasone, 50 ng/ml Ascorbate-2-phosphate, 1 mM sodium pyruvate, 1X ITS (6.25 μg/ml Insulin, 6.25 μg/ml Transferrin, 6.25 ng/ml Selenous acid), 50 μg/ml proline. The cell aggregates were allowed to grow for 3 weeks. Cell spheres were fixed with 4% paraformaldehyde for 24 hrs at room temperature and then were stained with 1% Alcian Blue (Sigma Aldrich, Germany).

### Statistical Analyses

In this study, umbilical cords were used from three different pregnancies. WJ-MSCs isolated from each umbilical cord were subjected to multi-lineage differentiation in three independent experiments. Statistical significance was estimated with a one-tailed Student’s t-test assuming equal variance and error is s.e.m. (*P *<* 0.05) [48].

## Results

### Cell isolation, proliferation and Characterization of WJ-MSCs

Umbilical cords were stripped of the cord blood vessels and Wharton’s jelly (WJ) matrixes were scabbed off into small pieces. WJ-MSCs were isolated using proteolytic enzyme digestion protocol and the cells were cultured in DMEM supplemented with 10% MSC-qualified FCS and 4 ng/mL bFGF. The freshly isolated WJ-MSCs grow as a flat monolayer and exhibit an elongated spindle-shape fibroblast-like morphology when cultured on polystyrene tissue culture plates (Fig 1B).

WJ-MSCs, seeded at a density of 1000 cells/cm^2^, did not show changes in growth rates at the first 5 days in culture. The following days 6–12, the cells demonstrated significant low proliferation rates, with an average accumulative population doubling rate (APDR) of 1.3 ± 0.88 and the average population doubling time (PDT) was 33.1 ± 5.7 hrs. Notably, a robust proliferation rates were recorded at culture days 13–26; the observed average APDR and PDT were 6.2 ± 1.7 and 8.1 ± 2.6 hrs, respectively (Fig 1C).

To assess the capacity and frequency of self renewal WJ-MSCs; cells (P2-P5) were seeded in two density groups (50, and 100 cells/cm2) and the new forming fibroblast colonies derived from single cells were assessed after 7 to 10 days. As shown in Fig 1D, lowering the cell density improved CFU-F. 50 cells/cm^2^ produced 13.4 ± 2.5 colonies which is significantly higher than the seeding density at 100 cells/cm^2^ that generated 9.8 ± 0.8 CFU-F.

Further, we performed proteomic analysis on the isolated WJ-MSCs. Data analysis has shown the expression of several stemness proteins including Prohibitin, Gelsolin, EF-hand calcium-binding protein RLP49, and Solute carrier family 2 (S1 Dataset).

### The expression of the CD- surface markers

**Fig 2:**
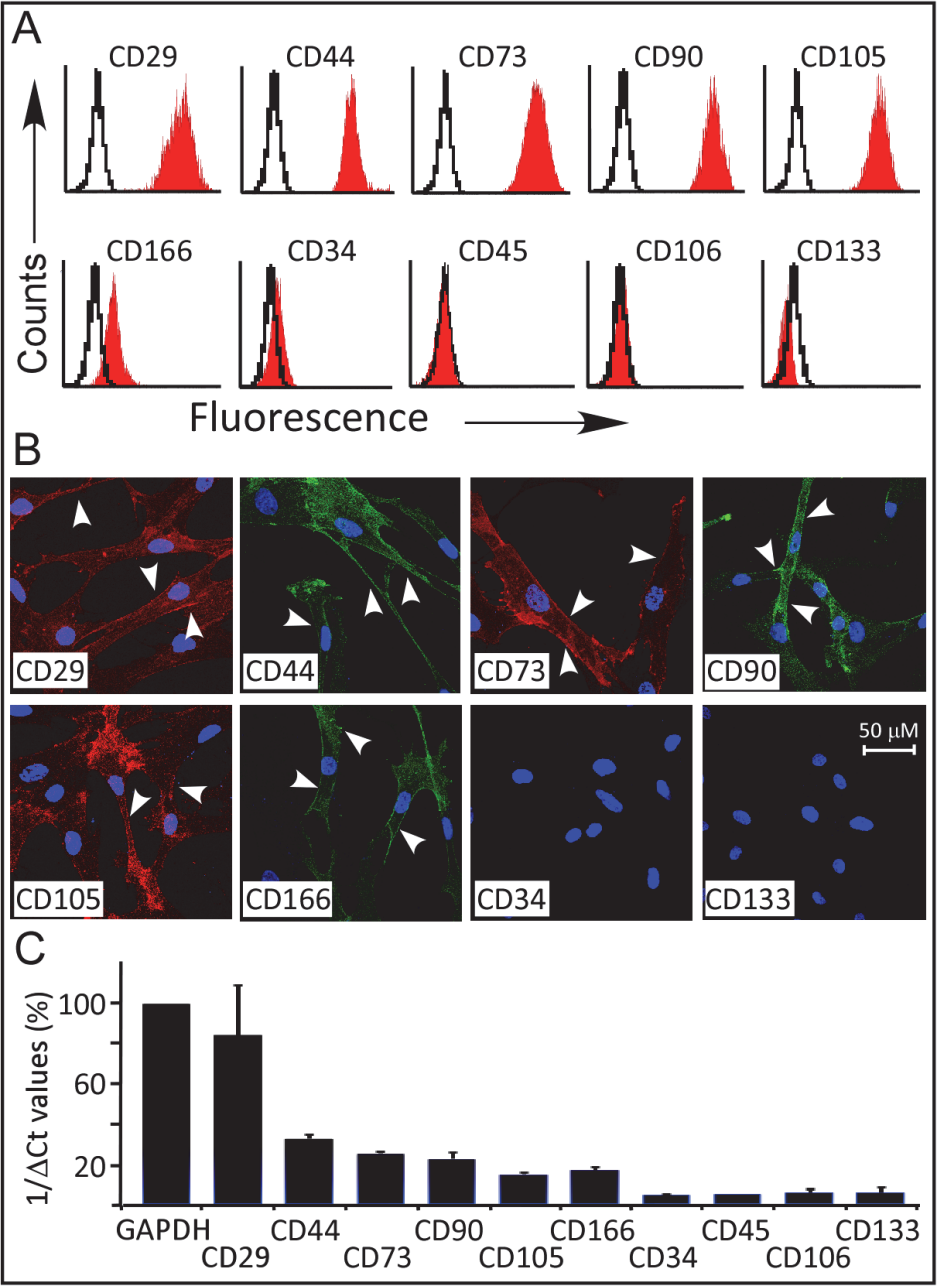
Flow cytometry, Immunofluorescence and qRT-PCR of WJ-MSCs. (A). Representative flow cytometry of WJ-MSCs (n = 3). Cells express CD29, CD44, CD73, CD90, CD105, and are negative for the hematopoietic (CD34 and CD45) and endothelial (CD106 and CD133) markers. Black open histogram indicates controls signal; red shaded histogram represents positive reactivity with the indicated antibody. (B) Confocal laser images of Immunofluorescence using APEX-labeling system for conjugating primary antibodies; CD29-Alexa Fluor 594, CD34-, CD44-, CD90- and CD133- Alexa Fluor 488. CD73-PE and CD105-PE were manufacturer labeled. 600X magnifications (C) qRT-PCR of the prospective markers for RNA isolated from undifferentiated WJ-MSCs cells, values were expressed as a percentage relative to 1/dCt of GAPDH gene.

Flow cytometry immune profiling, cell passage 3–5, revealed positive expression (>95%) for the putative mesenchymal stem markers CD29, CD44, CD73, CD90, CD105. The endothelial surface markers, CD106 and CD133, and the hematopoietic stem marker CD34 showed negative antigenic reactivity. While and the monocyte-macrophage CD45 marker showed minor reactivity (<5%), suggesting a minor contamination with hematocytes (Fig 2A). The expression of the surface markers was also observed by immunofluorescence analysis using anti-human CDs antibodies as described in Fig 2B (and S1 Fig). Confocal laser microscopy images show the cell membrane localization of the tested surface markers (arrowheads).

In accordance with the flow cytometry data, real time quantitative reverse transcriptase-polymerase chain reaction (qRT-PCR) analysis demonstrated statistically significant high gene expression levels of the integrin marker CD29 (82% relative to GAPDH). The expression levels of the matrix markers (CD44 and CD105), CD73 and CD90 were 15%- 38% relative to that of GAPDH. Low RNA expression (<4% relative to GAPDH) was detected for the hematopoietic and endothelial markers (Fig 2C).

### Multi-lineage differentiation capabilities of WJ-MSCs

According to ISCT guidelines, WJ-MSCs should exhibit the multi-lineage differentiation potential (adipogenic, osteogenic and chondrogenic), in order to be legitimate as functional MSCs. We studied the tri-lineage differentiation potential of WJ-MSCs and characterized the cellular markers in time dependent fashion. The differentiation potential was limited to cell passages 3–5.

#### Adipogenic differentiation

**Fig 3:**
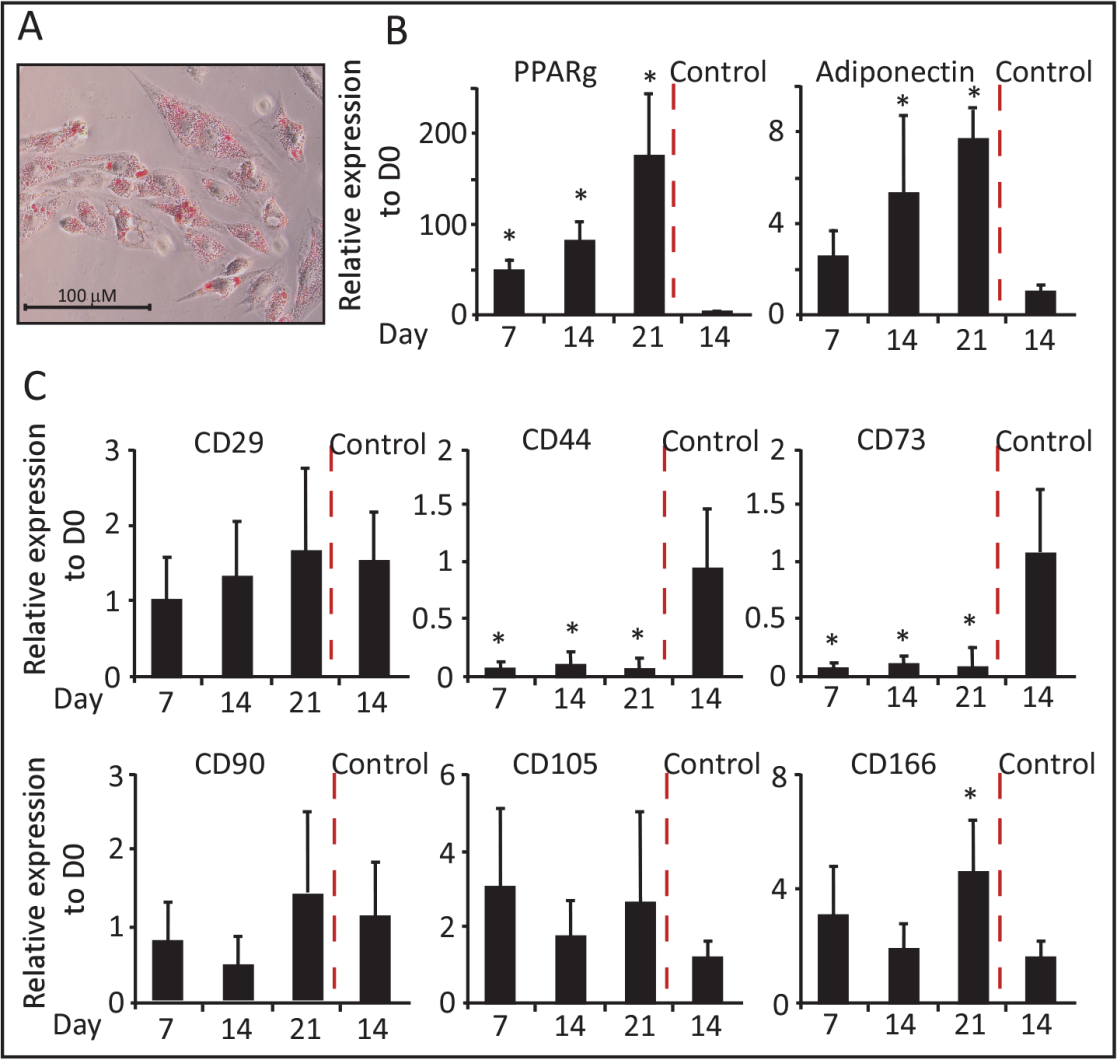
Adipogenic differentiation of WJ-MSCs. (A) Representative staining of adipocytes with Oil Red-O stain (200X). (B) and (C) qRT-PCR was performed after the induction of adipogenic differentiation of WJ-MSCs. Results for all panels were expressed as fold-change relative to Day 0—undifferentiated WJ-MSCs (n = 3, *p < 0.05).

In order to investigate the potential of WJ-MSCs differentiation into adipocytes; cells were treated with IBMX, dexamethasone and insulin for a period of three weeks. Cells were harvested at days 7, 14 and 21 for RNA extraction and qRT-PCR analysis using specific primers for the studied markers (Table 2). On Day 21 of differentiation, adipocytes were detected by Oil Red-O positive staining of lipid droplets (Fig 3A). At molecular level, qRT-PCR analysis demonstrated a significant time course dependent increase in the expression of adipogenic markers peroxisome proliferator-activated receptor gamma (PPARg) and adiponectin (Fig 3B), as compared to control-treated cells, which did not differentiate efficiently into adipocytes.

**Fig 4:**
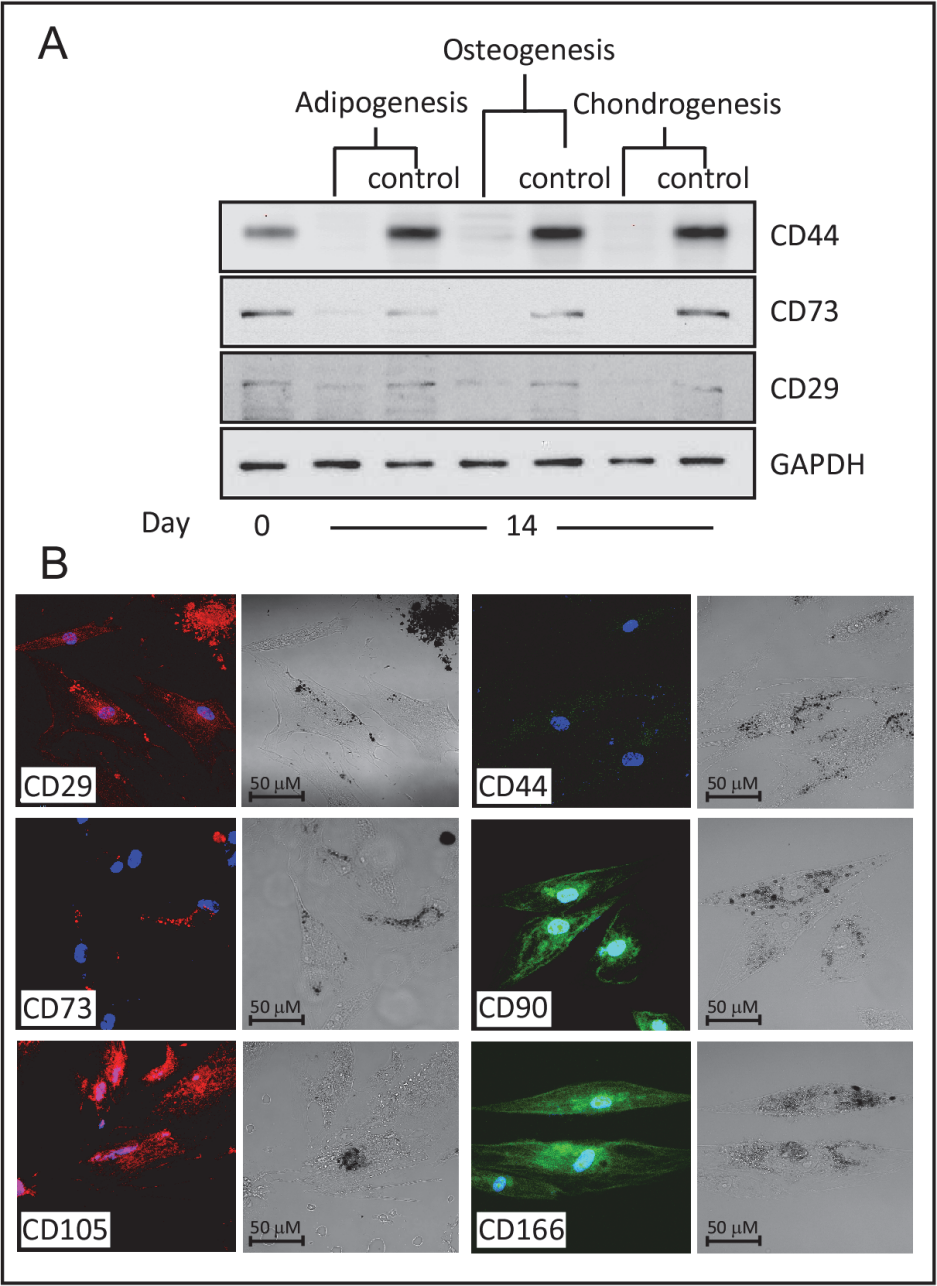
Protein expression of CD-markers during multi-lineage differentiation. (A) Western Blot analysis of undifferentiated (D0) and multi-lineage differentiated WJ-MSCs (D14). (B) Adipogenic differentiation of WJ-MSCs, immunofluorescence images using Confocal Laser microscopy (400X magnification). Primary antibodies were conjugated as described in Material and Methods. CD29-Alexa Fluor 594, CD44-, CD90- and CD166-Alexa Fluor 488, CD-73- and CD105-PE. Oil Red-O staining was observed as black dots using monochrome digital camera (phase contrast images) or red dots using 633-nm Laser beam.

To determine the authenticity of the stemness markers that characterize MSC, we studied the gene expression of these markers during the differentiation process. Interestingly, the transcript level of CD29 was equivalent to that of undifferentiated cells and day 14-control cells (Fig 3C). The expression of CD105 and CD166 showed a non significant 2–4 fold increase as compared to undifferentiated cells. The expression of CD90 was moderately reduced in the first two weeks, but then elevated at equivalent levels to that of the controls. Both CD44 and CD73 gene expression was dramatically reduced though out the course of differentiation (Fig 3C). In accordance with the gene expression analysis, a similar expression pattern of proteins from total cellular extracts of WJ-MSC- undifferentiated (D0) versus differentiated into adipocytes was detected (Fig 4A). Western Blot analysis, using specific antibodies against the CD markers, revealed a dramatic reduction in protein levels of CD44 and CD73, whereas the CD29 protein was sustained during adipogenesis.

The protein expression of the CD-markers was also visualized using immunofluorescence images post adipogenic differentiation of WJ-MSCs (Fig 4B), which was marked by Oil Red-O staining (observed as black dots in phase contrast images using monochrome camera, and as red spots under 633-nm Laser ray, e.g. CD73 images, Fig 4B). Unlike CD29, CD90, CD105 and CD166; the cell membrane expressions of CD44 and CD73 were not detected (Fig 4B), supporting the data obtained from molecular studies.

#### Osteogenic Differentiation

**Fig 5:**
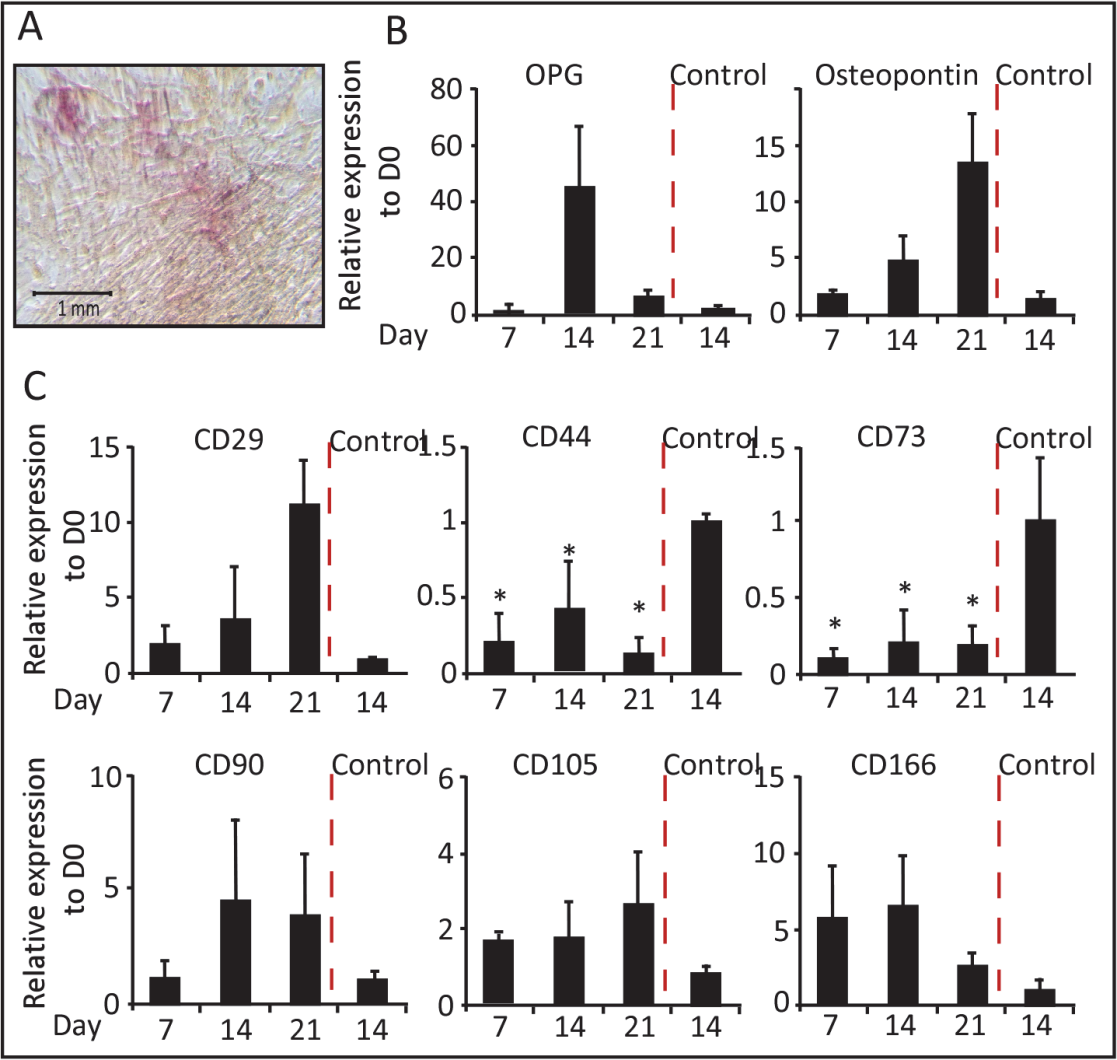
Osteogenic differentiation of WJ-MSCs. (A) Representative staining of osteoblast with alkaline phosphatase reactivity staining (100X). (B) and (C) qRT-PCR was performed after the induction of osteogenic differentiation of WJ-MSCs. Results for all panels were expressed as fold-change relative to Day 0—undifferentiated WJ-MSCs (n = 3, *p < 0.05).

The osteogenic differentiation of WJ-MSCs was determined by culturing them in media containing dexamethasone, β-glycerophosphate and ascorbate as described in Material and Methods section. RNA was extracted at days 7, 14 and 21 during the differentiation time course. On day 21 of differentiation, cells showed remarkable changes in cellular morphology and were stain positive for alkaline phosphatase activity (Fig 5A) and von Kossa staining of calcified bone matrix (data not shown). qRT-PCR analysis showed that the expression of osteoprotegerin (OPG) and osteopontin transcripts increased during the differentiation, almost 50-fold by day 14 and 15-fold by day 21, respectively (Fig 5B).

Similar to our observation during adipogenesis; CD29 and CD90 gene expressions were gradually increased during the differentiation time course and peaking at day 21. Whereas, CD105 and CD166 showed constant gene expression with 2 to 5-fold enhancement compare with control cells. On the other hand, CD44 and CD73 transcripts levels were 50 to 90% lower than the transcript level of control cell lines (Fig 5C). Subsequent Western blot analysis showed absences of CD44 and CD73 expressions, but not CD29, during osteogenesis relative to controls (Fig 4A).

#### Chondrogenic Differentiation

**Fig 6:**
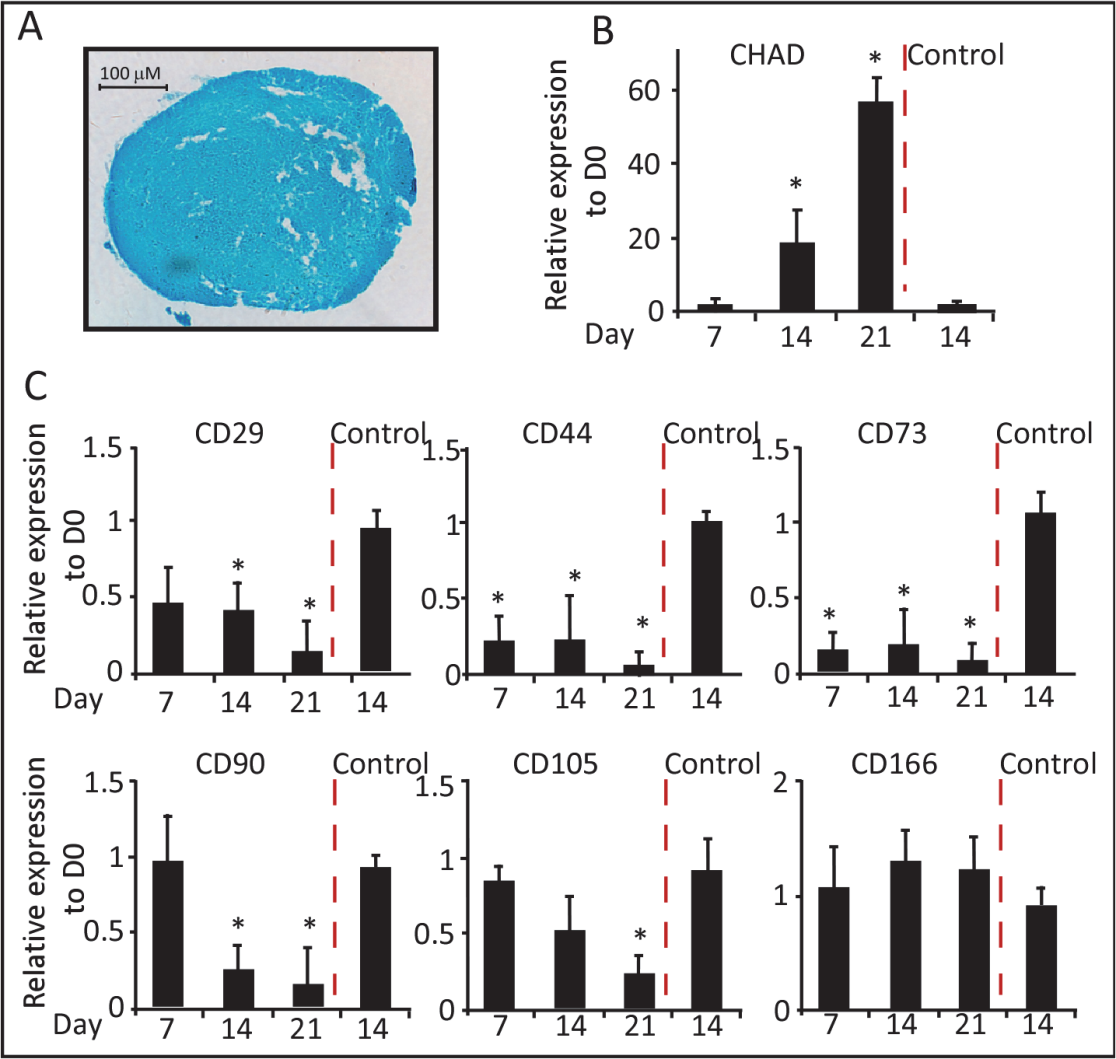
Chondrogenic differentiation of WJ-MSCs. (A) Representative staining of chondrocytes with Alcian Blue staining (200X). (B) and (C) qRT-PCR was performed after the induction of chondrogenic differentiation of WJ-MSCs. Results for all panels were expressed as fold-change relative to Day 0—undifferentiated WJ-MSCs (n = 3, *p < 0.05).

Chondrogenic differentiation was induced by aggregating WJ-MSCs in a serum free media containing TGF-β3, dexamethasone and ITS; for a period of three weeks. Following, two days culture in canonical tubes, the cell pellets lost attachment and formed spherical aggregates which increased in size indicating the production of extracellular matrix. Cell aggregates were harvested at days 7, 14 and 21 for gene expression. On day 21 of differentiation, cell aggregates were stained with Alcian blue a marker for glycosaminoglycans in cartilages (Fig 6A). qRT-PCR analysis of the chondrogenic marker chondroadherin (CHAD); showed a 20- and 60-fold increase in RNA expression at days- 14 and 21 of differentiation, respectively (Fig 6B).

Different gene expression patterns were detected for the stemness markers during chondrogenesis. Relative to the control cells, CD29 and CD90 transcripts were reduced after 7- and 14- days of chondrogenic induction, respectively (Fig 6C). The RNA levels of CD166 were maintained; while those of CD105 were gradually reduced during the differentiation time course. Similar to the other lineages, the expression levels of CD44 and CD73 RNAs were noticeably reduced as compared to undifferentiated cells (Fig 6C). These data were further supported using Western blot analysis, the protein expressions of CD29, CD44 and CD73 were noticeably reduced in response to chondrogenic differentiation (Fig 4B).

## Discussion

MSCs have proven to represent a major hope for cell-based therapy and tissue engineering applications, as these cells possess the capacity for self-renewal and multi-lineage differentiation potentials [54, 55]. In addition, MSCs can be used for clinical applications due to their immunomodulatory properties [56]. MSCs are isolated from several postnatal and extra-embryonic tissues, which are discarded as medical wastes and thus do not provoke technical or ethical concerns [52, 57]. Currently, similar to other MSCs, it is well established that WJ-MSCs express a wide range of surface antigen markers including CD29, CD44, CD73, CD90, CD105 and CD166; and lack the expression of the hematopoietic or endothelial markers [58]. However, in WJ-MSCs, there are no studies tracing the expression profile of these markers during the differentiation processes. In this study, we report the isolation and characterization WJ-MSCs that habitat the gelatinous portion of the umbilical cord. Further, we have elucidated the molecular profile of the commonly used stemness surface markers associated with WJ-MSCs differentiation.

WJ-MSCs displayed robust proliferation abilities. The growth curve of isolated cells revealed a characteristic pattern. In the first 12 days, cells showed a slow proliferation profile and then entered the logarithmic growth phase which continued for 14 days. It is reputable that self renewal and high proliferation capacity are critical for MSCs to maintain stemness characteristics during *in vitro* culturing [59]. Using the isolated WJ-MSCs, a systematic evaluation of the effect of plating density on CFU-F was performed. The cell seeding density was negatively correlated with the observed CFU-F frequency, suggesting a possible paracrine signaling or metabolic products that may reduce clonogenic frequency or down-regulate WJ-MSCs proliferation [60, 61].

In addition, we utilized the proteomic approach to further characterize the stemness authenticity of the isolated WJ-MSCs. Mass spectroscopy analysis revealed that these cells express proteins that have been previously identified to be stem cell related [25, 62–64]. With particular interest, two prohibitin proteins were identified PhB1 and PhB2; the former is reported to be crucial for the regulation of embryonic stem cells homeostasis and differentiation [65]. On the other hand, Gelsolin has been recognized as an important checkpoint in the differentiation of MSCs under the regulation of TGF-β [66].

In according with previous reports, we found that the undifferentiated WJ-MSCs co-express the stemness markers CD29, CD44, CD73, CD90, CD105 and CD166. qRT-PCR analysis revealed that CD29 is much more strongly expressed as compared to the other markers. Interestingly, the expression profile of these markers showed a distinctive pattern during the multi-lineage differentiations.

The transcripts of the transmembrane proteins integrin β1 subunit (CD29) and the Thy-1 (CD90) were found to be up-regulated and down-regulated upon the onset of osteogenic and chondrogenic lineages, respectively; with more prominent expression profile after three weeks of differentiations. CD29 and its modulator ICAP-1 are required for osteoblast proliferation and differentiation explaining the observed increase in its expression during osteogenesis [67]. Our results confirm previous reports observing down regulation of CD90 during differentiation in human bone marrow MSCs (BM-MSCs) [41, 68, 69], and synovium derived MSCs [70]. We observed a steady upregulation of CD29 expression during adipogenic differentiation, but CD90 transcripts were reduced, supporting previous reports indicating that CD90 is negatively correlated with adipogenesis [71]. CD105, also called endoglin, is a type I membrane glycoprotein receptor that is a part of the transforming growth factor β (TGFβ) receptor complex. The expression of CD105 was moderately upregulated during adipogenic and osteogenic; but not chondrogenic differentiation. Treatment with TGFβ induces chondrogenesis and is associated with CD105 low protein expression in human bone marrow MSCs (BM-MSCs) cultured on micromass and alginate systems [68, 69, 72].

CD166 with adhesive properties belongs to the type I transmembrane glycoprotein as a member of the immunoglobulin super-family of protein. Similar to the previously reported proteomic studies on human BM-MSCs [35], we found that the expression of CD166 is upregulated during adipogenic and osteogenic differentiations. No changes in the expression profile of CD166 were detected in cells undergoing chondrogenesis. These results contradict a previous data showing that CD166 protein is down-regulated during chondrogenic differentiation in BM-MSCs. The discrepancy can be explained by the differences in BM- versus WJ-MSCs, or explained by possible post translational regulations that causes lower rates of protein translation compare to RNA transcription.

Interestingly, the expression levels of CD44 and CD73 were dramatically reduced during the multi-lineage differentiation process. Protein studies have shown that the hyaluronan receptor, CD44, is down regulated during chondrogenesis and osteogenesis in human BM-MSCs [35, 69]. CD73, on the other hand, was reported to be slightly increased in human BM-MSCs undergoing adipogenic and osteogenic differentiation [35].

Our focus on the commonly used MSCs markers and tracking their expression profile during multi-lineage differentiation may provide key insights on WJ-MSCs signature. Currently, there is no single molecule or protein that can be used for WJ-MSCs identification and isolation. Interestingly, the commonly used positive markers are selected to include surface antigens that are absent from most hematopoietic cells, which might be beneficial to isolate BM-MSCs, but not MSCs from other tissues. The existence of CD29, CD90, CD105 and CD166 transcripts in terminally differentiated cells, suggests that these markers may not be ideal markers to identify and isolate WJ-MSCs, since the later cells are impeded in a tissue layer rich in adipogenic-, smooth muscle- and vascular tissues. On the contrary, the low expression levels of CD44 and CD73 during multi-lineage differentiation imply that these two markers have low cellular abundance; at least in part, at the tri-lineages studied. Thus, CD44 and CD73 are reliable stemness markers for WJ-MSCs, since their expression is associated with undifferentiated WJ-MSCs only.

## Supporting Information

S1 Dataset Proteomic analysis of undifferentiated WJ-MSCs. Total proteins were extracted and digested. The generated peptides were subjected for MS/MS analysis. The generated spectra were acquired in a data-dependant acquisition mode selecting top 20 spectra. Raw data files were analyzed using Maxquant 1.3.0.5 software using Sequest and Mascot search engine against the Homo sapiens International Protein Index (IPI) protein sequence database version 3.68 (European Bioinformatics Institute, United Kingdom). Stem cell specific proteins which were previously reported are list and referenced.
(XLSX)

S1 Fig Immunofluorescence analysis for undifferentiated WJ-MSCs. Cells were incubated with antibodies directed against the individual surface markers. Confocal laser images of Immunofluorescence using APEX-labeling system for conjugating primary antibodies; CD29-Alexa Fluor 594, CD34-, CD44-, CD90- and CD133- Alexa Fluor 488. CD73-PE and CD105-PE were manufacturer labeled. Phase contrast images 600X magnifications. Nuclei are stained with Hoechst. The CD-markers proteins are located at the cell member as observed by the Immunofluorescence.
(TIF)
