# Supplementary figures and images for: Multi-Lineage Differentiation of Human Umbilical Cord Wharton’s Jelly Mesenchymal Stromal Cells Mediates Changes in the Expression Profile of Stemness Markers
Source: PLoS One. 2015 Apr 7;10(4):e0122465. doi: 10.1371/journal.pone.0122465 (PMC4388513; doi:10.1371/journal.pone.0122465)

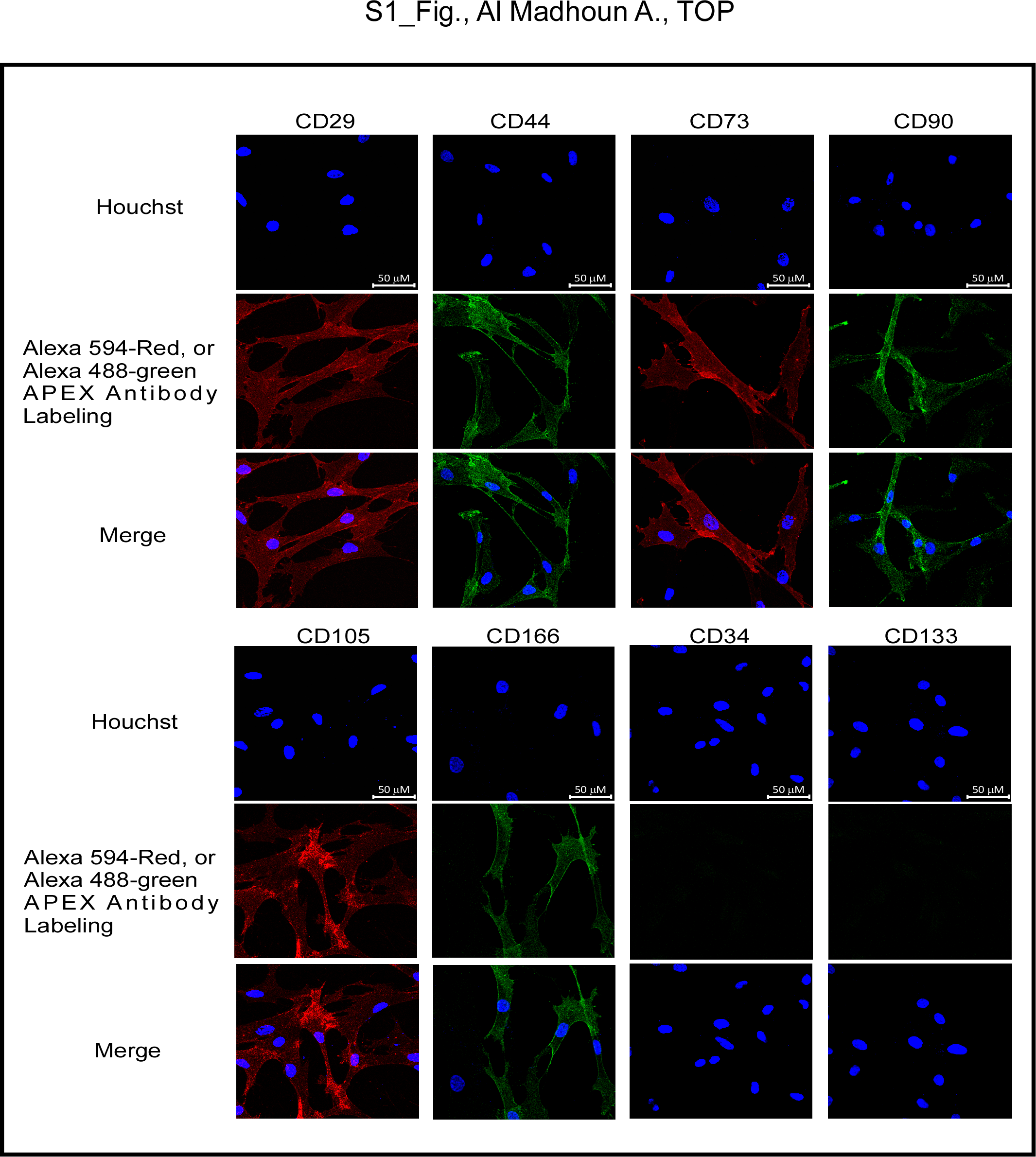

Supplement: S1 Fig — Cells were incubated with antibodies directed against the individual surface markers. Confocal laser images of Immunofluorescence using APEX-labeling system for conjugating primary antibodies; CD29-Alexa Fluor 594, CD34-, CD44-, CD90- and CD133- Alexa Fluor 488. CD73-PE and CD105-PE were manufacturer labeled. Phase contrast images 600X magnifications. Nuclei are stained with Hoechst. The CD-markers proteins are located at the cell member as observed by the Immunofluorescence. (TIF) [file pone.0122465.s002.tif]
